# Emotional responses in archival work

**DOI:** 10.1007/s10502-023-09419-5

**Published:** 2023-06-30

**Authors:** Cheryl Regehr, Wendy Duff, Jessica Ho, Christa Sato, Henria Aton

**Affiliations:** 1 Factor-Inwentash Faculty of Social Work, University of Toronto, Toronto, Canada; 2 Faculty of Information, University of Toronto, Toronto, Canada

**Keywords:** Emotions, Workplace, Archivists, Diary research, Archival organizations

## Abstract

Building on previous work investigating the impact of exposure to (a) records with traumatic potentialities and (b) interactions with donors and community researchers whose suffering is documented in the archives, this study sought to better understand emotional aspects of archival work. Using a diary research methodology, 15 archivists engaged in diary keeping for approximately four months. What emerged was a broad set of events and experiences that triggered a wide range of emotional responses arising from archival work. This included: pre-existing emotional states and characterological traits; emotional exchanges in the workplace with colleagues and others; emotional demands of the work (including emotion work and emotional labour); team and leader interactions arising from group tasks and leader behaviour; and organizational policies, climate, resources and demands. This broader set of interactional factors forms the foundation on which traumatic and other troubling events are encountered. Future research must consider the nature of archival organizations and interactions within them that contribute to the overall working experience. In addition, archival organizations need to take responsibility for creating a culture that demonstrates respect and appreciation for workers, acknowledges the interpersonal challenges of the work, and provides supports for archivists who are shouldering the challenges.

## Introduction

A relatively recent focus of scholarly work in the field of archives has surrounded emotional trauma in archivists arising from the engagement with records that immortalize human suffering (Nathan et al. 2015; Regehr et al. 2023; Sexton 2019; Sloan et al. 2019). That is, “archivists tasked with designing and managing information systems that document horrific events in human history” (Nathan et al. 2015), p 92). Digital archivist Elvia Arroyo-Ramirez (2021) identified the experience of “*suspended grief,* or grief experienced, witnessed, and re-lived throughout an archive… when processing collections about traumatic events and experiences.” Jo Moran-Ellis (1997) described a sense of “pain by proxy” when reflecting on her experiences as a researcher working with records of child sexual abuse. Others have identified intrusive thoughts, disturbed sleep, and feeling compelled to share disturbing cases with unwilling relatives and friends as researchers reviewing suicide records in a coroner’s office for a research project (Fincham et al. 2008).

Research with archivists also demonstrates that interacting with, and supporting, traumatized individuals—donors and community researchers—whose lives are reflected in the archives can be rewarding, but also emotionally challenging. Jennifer Douglas and Alexandra Alisauskas (2021), for instance, suggest that creating archives in collaboration with bereaved family members contributed to “grief work”, validating that a life existed or experience occurred, and presenting a way surviving family members could express love. In this way, Geoff Wexler and Linda Long contend that archivists serve as “guardians of a personal legacy” (Wexler and Long 2009 p 485), a role bestowed on them by donors of deeply personal records (Regehr et al. 2022). Previous research has also suggested that community researchers who seek the assistance of archivists may be survivors of trauma, such as in the case of Indigenous researchers who are survivors of the residential school system in Canada (Regehr et al. 2022). These researchers may be seeking validation of their experiences and memories, Michelle Caswell, Marika Cifor and Mario Ramirez have described this as “to suddenly discover yourself existing” (Caswell et al. 2016, p 56). In these cases, archivists may be in the role of bearing witness to disclosures of abuse and suffering and as a result experience their own emotions of shock and horror and profound senses of anger, sadness and at times despair (Regehr et al. 2022).

Research with a wide variety of occupational groups, more recently including archivists, has studied individual, event and organizational factors that contribute to an individual’s susceptibility or resilience to distress when exposed to traumatic stimuli in the workplace. This has included factors that are specific to the individual, including previous trauma exposure, resilience, optimism and coping styles (Collins 2007; Dagan et al. 2015; Douglas et al. 2019; Jenkins and Baird 2002), and the existence of a personal network of social supports (Ennis and Home 2003; Marmar et al. 2006; Regehr 2009). Factors related to the traumatic exposure itself can influence trauma responses including: the nature of the content (Birze et al. 2023; Nathan et al. 2015; Regehr et al. 2022); the form it takes (Birze et al. 2023; Polak et al. 2019), the intensity and length of exposure (Bober and Regehr 2006; Resnick et al. 1992), and the degree to which it is personally meaningful (Douglas et al. 2022; Regehr et al. 2022, 2002). The nature of the organizational environment has also been found to mitigate emotional response to workplace trauma exposure including factors such as workload, organizational climate, social support and supervision (Bell et al. 2003; Carpenter et al. 2013; Davis-Sacks et al. 1985; McFadden et al. 2015; Sloan et al. 2019). When an organization is perceived to value the contribution of workers and support their well-being, workers are better able to cope with the emotional demands of the job and have better work–home life balance (Alfandari et al. 2022; Elpers and Westhuis 2008).

### The current study

The current study sought to build on previous work investigating the impact of exposure to (a) records with traumatic potentialities (Sexton 2019) and (b) interactions with donors and community researchers whose suffering is documented in the archives. Using a diary research methodology, 15 archivists engaged in diary keeping for approximately four months, eight of whom participated in a previous interview study examining response to potentially traumatic events. Arising from previous research (Regehr et al. 2022, 2023), we expected that diary entries would focus on traumatic exposures, and subsequent emotional responses to these exposures. What emerged, however, was a much broader set of events and experiences that triggered a wide range of emotional responses arising from archival work. This included emotions brought to the workplace from other personal experiences; emotions arising from interactions with others including co-workers and managers; emotions arising from the work with records, community researchers, and donors; and emotions arising from the nature of the workplace itself. The diary data regarding the nature of experiences that provoked emotions and the nature of emotions encountered caused us to expand beyond literature and conceptualizations of *trauma* in the workplace, and venture into the literature related to a wider set of *emotions* in the workplace.

The literature reveals that emotional responses in the workplace have both immediate- and long-term effects, not only on the individual experiencing the emotional reactions, but also others, and the organization itself. For instance, negative emotions in the workplace have been associated with counterproductive behaviours (Matta et al. 2014), emotional exhaustion (Portoghese et al. 2017) and burnout (Miller et al. 2007), and reduced overall performance (Weiss and Cropanzano 1996), whereas positive emotions are associated with having a sense of meaning, achievement (Miller et al. 2007), and well-being (Junça Silva et al. 2022), as well as engagement (Junça Silva et al. 2022), job satisfaction, and commitment to the organization (Ashkanasy and Dorris 2017). This paper reports on this expanded view of emotions in the archival workplace and a reconceptualization of one model (Ashkanasy 2003) for understanding emotions in the workplace.

### A model for understanding emotions in the workplace

**Fig. 1:**
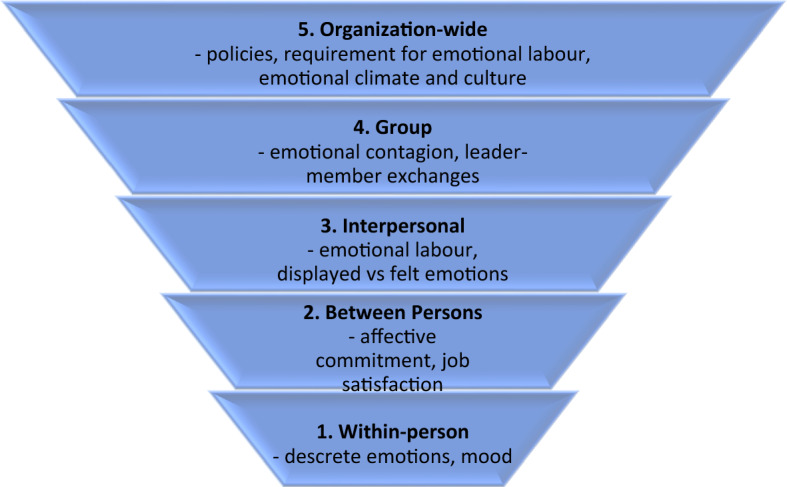
Five-level model of emotions in the organizations. Summarized from (Ashkanasy 2003)

Interest in emotions in the workplace arose in the 1920s and 1930s with the development of new fields of study, industrial and organizational psychology which focussed among other things on individual differences among workers with respect to job satisfaction and motivation, and subsequently how this impacted performance and productivity. Researchers in the 1960 and 1970s by contrast focussed on characteristics of the organization and the work, and the manner in which factors such as personal agency and organizational justice affected both workers and work (Weiss and Brief 2001). Integrating these various perspectives, organizational psychologist Neil Ashkanasy proposed a five-level model for understanding emotions in the workplace ranging from that which resides within a particular employee; to that which represents interactions between individuals and within workplace groups; to that which are organization-wide, arising from the policies and culture of the organization (Ashkanasy 2003). This model is depicted in Fig. 1.

In Ashkanasy’s model, level 1 (within-person) refers both to the individual’s emotional response to specific workplace events and the individual’s overall mood which affects the perception of events, meaning attributed to events, that contribute to emotional response and resulting behaviours. The second level (between persons) refers to dispositional aspects of the individual and their emotional intelligence in interacting with others. This is then understood to influence the individual’s job satisfaction and affective commitment to the organization. Level 3 (interpersonal) is described by Ashkanasy as the emotional exchange in interactions. This includes the notion of emotional labour required in the job, that is, when the emotion that the employee is required to convey by virtue of their role is inconsistent with that which is felt (Hochschild 1979, 1983). Level 4 (groups) is the emotional climate and rules of the group, and the way in which the leader manages emotional exchanges and models emotional expectations. Finally, level 5 (organization-wide) is the overall emotional climate of the organization, and policies which govern emotional responses (Ashkanasy and Dorris 2017). This model was used as a framework for organizing the data arising from diaries kept by archivists in this study. However, our analysis had led to a modification of the model to better reflect contextual factors in the workplace that influence emotional responses of archivists during their working days. This modification can be found in the discussion section.

### Affective events theory (AET)

“Affective Events Theory focusses on the structure, causes, and consequences of affective experiences at work” (Weiss and Cropanzano 1996, p 11). From this perspective, affective levels fluctuate over time and are influenced by a variety of factors some of which are internal to the individual, including pre-existing mood state and disposition; some of which are environmental, such as structure, policies and expectations of the organization; and some of which arise from interactions or “dynamic transaction[s]” (p 39) between the individual and the environment—including other members of the organization. Howard Weiss and Russell Cropanzano suggest that in the short term, employee behaviour can be tied to emotional response to specific “emotion generating events” (p 31) in their workplace environment. In the longer term, emotional responses to affective events contribute to, or detract from, work-related attitudes (such as job satisfaction and commitment) and behaviours (overall performance).

Unlike the body of research that focuses on traumatic events in the workplace that might lead to post-traumatic stress reactions (APA 2013), AET explores the impact of daily micro-events (DME) in the workplace (Junça-Silva et al. 2023; Junça Silva et al. 2022), what Lazarus (Lazarus 1986) earlier described as daily hassles that frustrate people at work. Often these events focus on issues arising from interactions with fellow employees or team members, managers or directors, other teams within the organization, or overarching organizational policies (Chacko and Conway 2019). But they also include technical and other frustrations, for instance in our increasingly technical world, lost connectivity to networked resources (Chacko and Conway 2019), increased complexity in steps to complete certain activities (Frintner et al. 2021; Privitera et al. 2018), or systems that do not allow for sufficient flexibility to creatively address challenges. On the positive side, DME can also have positive effects on emotion (Rueff Lopes et al. 2017) resulting in “daily uplifts” which can contribute to increased work engagement and well-being (Junça Silva et al. 2022).

### Diary methods in workplace emotions research

While perhaps less commonly used than interviews and focus groups (Milligan and Bartlett 2019), there is an established practice of using diary methodology to explore emotions arising from affective events in the workplace (Chacko and Conway 2019; Rueff Lopes et al. 2017). For instance, diary methods have been used to explore emotions associated with labour relations (Holman 2016; Poppleton et al. 2008; Tschan et al. 2005), workplace bullying (Rodríguez-Muñoz et al. 2022), work-related stress (Bono et al. 2007; Clarkson and Hodgkinson 2007; Sonnentag et al. 2008), the impact of workplace violence (Portoghese et al. 2017) and events that are perceived to be unfair (Barclay and Kiefer 2019; Matta et al. 2014). Solicited qualitative diary studies have also been used to study information seeking behaviours of archive and library users (Colosimo and Badia 2021; Kuhlthau 1991; Melssen 2012; Toms and Duff 2002), and emotional labour in library staff (Matteson et al. 2015).

Unlike studies of archival diaries (see, for example, Allport 1942; Thomas and Znaniecki 1920) in which research is conducted on pre-existing unsolicited diaries, the diary method of qualitative research often calls for solicited diaries constructed to evoke answers to specific research questions. Solicited diaries capture data from participants in situ, allowing for more spontaneous, immediate and naturally contextualized data gathering as compared to traditional, retrospective research methods (Hyers 2018). Each entry in a diary study “is sedimented into a particular moment in time: they do not emerge ‘all at once’ as reflections on the past, but day by day strive to record an ever-changing present” (Plummer 1983), revealing, for instance, that the manner in which individuals cognitively frame the causes and consequences of workplace events shift from day to day (Clarkson and Hodgkinson 2007). Thus, solicited diaries are an excellent means to study subjective experiences, emotions and affect in their everyday contexts.

## Method

A total of 15 archivists kept diaries for four months. While participants were offered the option of submitting copies of handwritten notes, audio content, or completing online forms, all submitted online forms (with the exception of one handwritten entry). Diary keepers also completed a demographic form and signed an informed consent form before completing their first diary entry. Each diary keeper was offered a $200 honorarium prior to recording their first diary entry. This study was approved by the Human Subjects Research Ethics Board of University of Toronto.

### Participants

Participants were recruited through two means: general invitations distributed through the Canadian archives listserve ARCAN-L (7 participants) and specific invitations to individuals who had participated in a previous study (8 participants). All participants worked as archivists, with job titles that included archives manager, archives coordinator and collections manager. Thirteen diary keepers worked full time, while two worked part-time. While all diary keepers indicated that their positions included working with records, the number of hours dedicated to this work varied from 50% (7 individuals) to 25% (1 individual). Working with archival researchers/users constituted more that 50% of their time for 4 participants, less than 25% of their time for 9 participants, and none of their time for 1 participant. Only 11 archivists worked with donors; 9 diary-keeper spent less than 10% of their work time occupied with donor-related tasks; and only 1 indicated more than 25% of their job involved this work. Other tasks ranging from less than 10% of their work to a high of 50% included staff management, meetings, committee work, website design, project work, software work, database design, outreach, teaching, metadata work, interpretation, access, acquisitions, working with colleagues, grant writing, budgets, exhibitions, and programming.

### Data collection

Guidelines provided to participants requested that diary keepers complete a diary entry each day that they had a meaningful emotional response after working with records, supporting researchers, or working with donors. For each event recorded, diary keepers were asked to describe: the event; who or what they were interacting with when the event occurred; the feelings and reactions they encountered; and, if they shared the event with others, what reaction they received. In addition, participants were asked to indicate which of a series of emotions or mood states they experienced (happiness, enthusiasm, optimism, anxiety, anger, irritation, depressed, bored, tired, relaxed, content, satisfied), rated as: not at all (0); a little (1); some (2); or a lot (3). The number of diary entries that each diary keeper recorded ranged from a low of 5 entries over the 4 months to a high of 67 entries, with an average of 19 entries per diary. The amount of information in each entry also varied considerably. The number of words per diary ranged from a high of 7571 words per diary to a low of only 395 words with an average of 3260 words per diary.

### Analysis

Diaries were subjected to line-by-line micro-analysis and coding, allowing researchers to interact with the data and examine emerging themes. The two senior researchers (one a professor of social work and one a professor of archival science) and two research assistants directly engaged in reading, analysing and coding of diaries (Strauss and Corbin 1998). Initial categorization was guided by the model for understanding emotions in organizations developed by Ashkanasy (2003). Within these broad categories, emerging themes and patterns were identified and categorized, followed by determining interrelationships between the categories in an iterative and reflexive manner (Ben-Ari and Enosh 2011). Further conversations among researchers and with other experts allowed for the clarification of concepts and integration into an emerging theory (Glaser and Strauss 1967).

## Results

**Fig. 2:**
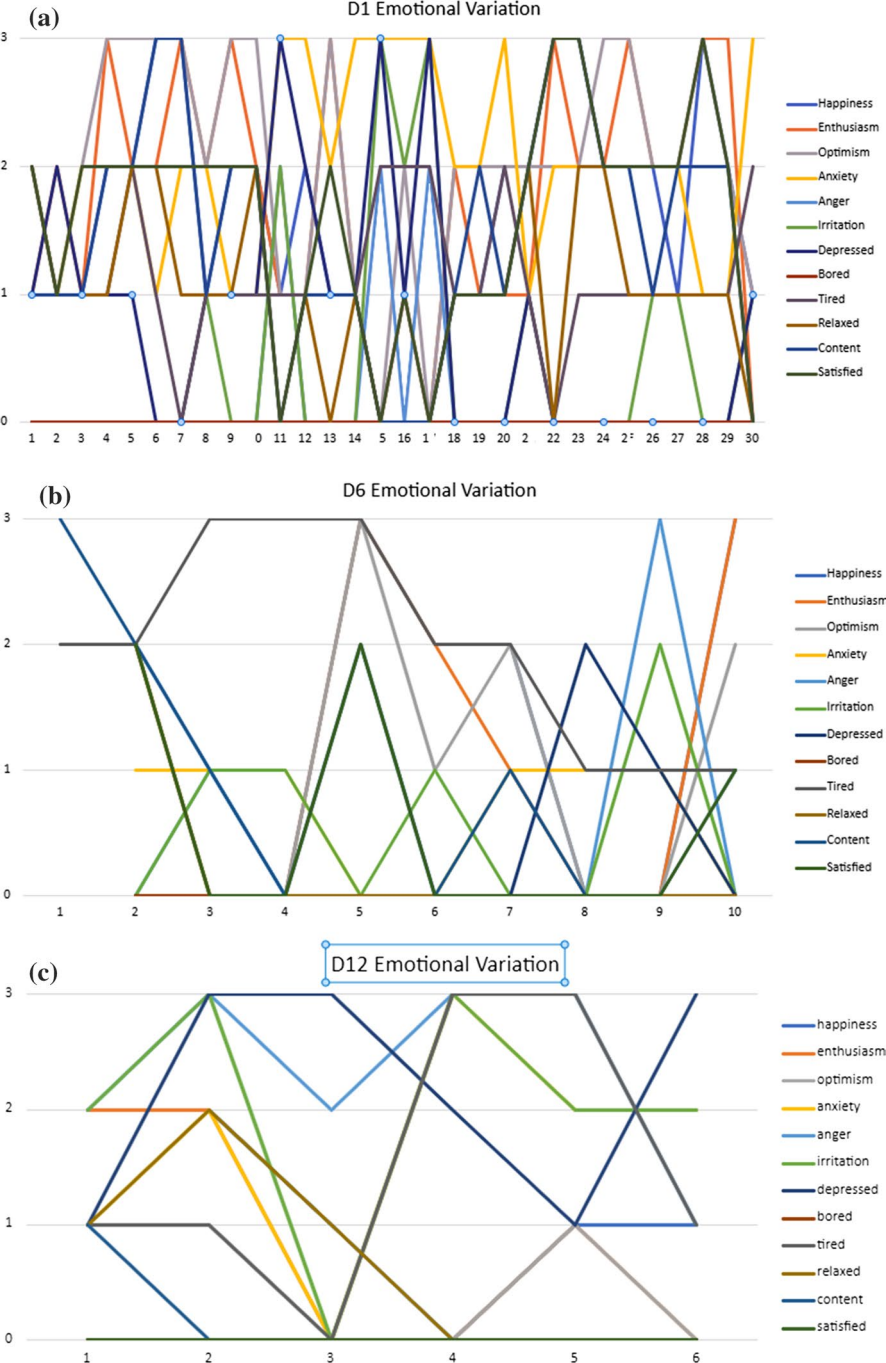
**a**–c Examples of emotional variation

While qualitative interviews have the benefit of collecting in-depth information around specific emotional encounters or experiences in the workplace, diaries allow researchers to see variations in emotion over a period of time. What is perhaps most striking in this diary study, is the variety of emotions identified by participants, which varied not only from entry to entry, but also co-existed on a particular day. Figures 2a–c depict specific emotions and emotional intensity identified by three participants. Participant 1, who created 30 diary entries, frequently reported high levels of enthusiasm, satisfaction, and optimism, interspersed with anxiety. Participant 6, who created 10 diary entries, expresses “a lot” of anger on one day, but happiness, optimism, and enthusiasm on others. Whereas participant 12 created 6 diary entries, 5 of which expressed “some” or “a lot” of anger.

**Fig. 3:**
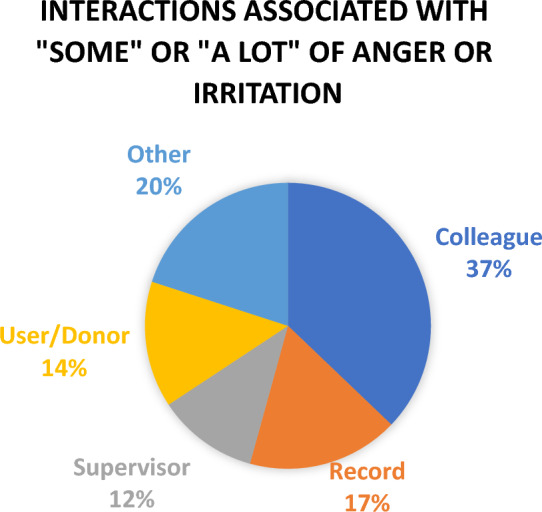
Interactions Associated with “Some” or “A Lot” of Anger or Irritation

**Fig. 4:**
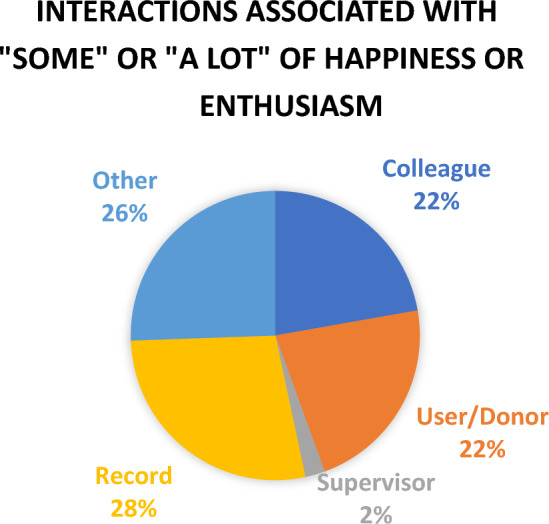
Interactions Associated with “Some” or “A Lot” of Happiness or Enthusiasm

Affective events (Weiss and Cropanzano 1996) that were associated with “some” or “a lot” of anger or irritation were most likely to involve other members of the organization including colleagues (37%) and supervisors (12%), rather than events arising from work with donors and users (14%) and records (17%). The other category in Fig. 3 includes: several members of the organization; individuals from other organizations; and other community members. On the other hand, affective events associated with happiness and enthusiasm most likely involved the nature of the work, with records and user/donors interactions representing 50% of the identified affective events. The other category in Fig. 4 most frequently reports interactions with members of the community. Supervisors were very infrequently associated with affective events associated with happiness and enthusiasm (2%).

Following a slightly modified approach to the five-level model of emotions in the workplace (Ashkanasy 2003), the remaining presentation of results is divided into the following sections: intra-individual emotions including personal issues brought to the workplace, pre-existing emotional states, and characterological traits; emotional exchanges in the workplace including interactions with colleagues; emotional demands of the work including emotional labour arising from working with troubling records, and working with researchers and donors; team and leader interactions including group tasks and leader behaviour; and organizational influences such as policies, climate, resources and demands.

### Intra-individual emotions

Central to understanding emotion in the workplace are differences in the person encountering various experiences (McFarlane and Yehuda 1996; Miller et al. 2007; Regehr 2018). First, individuals encounter the workplace with pre-existing emotional states arising from other aspects of their lives. For instance, one participant in the current study stated, “This is super personal, so I'll only say so much, but I've been having a rough time at home and have had some news from my partner today that is upsetting and I feel quite angry about it.” Secondly, individuals experience more pervasive emotional states that may arise from personal life experiences, or the job itself. For instance, two individuals identified pervasive negative feelings “Anxiety, overwhelm. It somehow feels normal in this job though.” “Just feeling the stress and feeling trapped and no control over my options.” Another identified pervasive positive feelings upon returning to work after an absence “it felt like a mixture of both hope and responsibility… and it felt like my heart felt full and warm when I came into the room.” This person further identified “The entire time, I kept being struck by the thought that I was so lucky to be able to do this kind of work: connecting to the records and the person who painstakingly made them… It was intense to feel so happy…”.

These pre-existing and pervasive states can influence worker’s interpretations of events encountered in other spheres, such as the workplace. For instance, anxiety is associated with an increased likelihood of interpreting ambiguous stimuli, such as facial expressions or ambiguous words as threatening (Blanchette and Richards 2003; Richards et al. 2002). Angry individuals tend to rely more on heuristics and stereotypes rather than consider a broader range of facts (Blanchette and Richards 2010). These pervasive or pre-existing mood states can also influence an individual’s appraisal of themselves, sometimes negatively. One participant observed their reactions to an affective event in the workplace: “Stress. Guilt. Fear that I had lost something through negligence. Regret that my actions might have caused this problem. Distrust of my own methodologies and memory. This emotional response is similar to what I have experienced in like circumstances.” Mood states can also impact positive self-appraisals as exemplified by the following quote: “satisfaction and confidence in my experience and ability. This sets me up nicely for getting intellectual control of one or two other complex areas… I feel good at my job.”

### Emotional exchanges in the workplace

Frequently participants in this study documented positive emotional exchanges with others, such as colleagues: “Our conversation was motivating and reassuring. I felt very happy and elated with enthusiasm”; and “I felt satisfied, happy and optimistic”. Another described how interactions with a colleague extended positive feelings: “I felt happy… It did not last long. I texted a colleague who works at another institution, and she congratulated me, so that extended the moment.” Several participants expressed positive feelings in interactions with researchers and donors, for instance: “I was overjoyed to hear their kind words”.

Addressing shared challenges with others provoked positive feelings: “I felt happy and optimistic because I felt valuable and had something to contribute… I experience the same or similar emotions in similar situations when I am able to help a colleague, another student, or a donor” (D8); “Gratitude for my colleague’s work and linguistic ability.” Another described sharing frustrations regarding workload with a colleague: “My colleague and I have a good relationship so it feels good to vent with her.” Another shared a mistake she made: “My colleague’s support was a great comfort… Her support makes me want to be better, more understanding or empathic.”

At other times participants described negative emotions arising from interpersonal encounters. One participant stated: “I still feel very angry, sad, irritated, and frustrated. The level of anger has lessened since yesterday, but my irritability, sadness, and frustration feels like it increased today.” (D7) Another recounted an emotional encounter with a colleague as follows:

“He accused me of making political maneuvers… and instructed me to have my defenses ready for retaliation… I was completely bewildered and upset, so much so that I started crying during the call, and had a panic attack immediately afterwards.”

However, while negative examples of emotional interaction with colleagues were reflected in diaries, many of our participants also described the joy of one-to-one interpersonal encounters. These positive relationships may serve as a buffer against negative affective events encountered at other levels.

### Emotional demands of work

In keeping with our original expectations regarding emotional implications of encounters with troubling records, and traumatized community researchers and donors, many of the diary entries related to these types of affective. Caswell and Cifor refer to “archivists as caregivers, bound to record creators, subjects, users and communities” (Caswell and Cifor 2016) (p 34). In this vein, one of the participants in this study indicated: “maybe my role as archivist (instead of gatekeeper or facilitator for information) is instead being a conduit of care, for the creator of records, those in the records, and those accessing the records”.

Participants described working with records involving systemic injustice, as well as those containing depictions of individual abuse and violence. They described emotions that ranged from shock, to anticipatory anxiety, to anger, to sickened, and to sadness. Examples of emotional experiences working with records include:

“I felt a certain anxiety each time I grabbed a file knowing full well that there was a good chance I would come across photographs that were difficult to look at.”

“Just seeing his name on the boxes makes me feel anger; anger that we are literally sheltering an abuser in our vault.”

Previous research has suggested that traumatic emotions or other negative emotions such as anger are not acknowledged or supported in the archival workplace, and thus they are often suppressed and experienced alone (Regehr et al. 2022).

In other cases, participants described emotional experiences when working with individuals who had endured hardship or traumatic experiences: “I experienced a range of emotions, including empathy for the difficult life experience of the Indigenous people who spoke to us”, and “Our donor is a survivor of the forced uprooting and dispossession of [people] during the Second World War… I had a lot of intense feelings of compassion, empathy, appreciation, and love for this donor.” These compassionate responses have previously been referred to as “emotional work” (Miller et al. 2007), p 325). Emotional work is central to the work of human service professionals who assist through establishing connections and listening to, and empathizing with, emotional stories of others.

Working with demanding community members and hiding feelings that might have emerged in the archivist is another form of response to affective events. One participant suggested: “I was definitely surprised [by the] sarcastic/negative response of the user… [but] my initial response of surprise/hurt dissipated when I realized what this person was trying to do… and I just brushed the email aside.” Another in reflecting on a message from a community member stated “Anxiety, irritation, and fear. I managed to send a cursory response in about 30 min and that alleviated some of the pressing anxiety”.

Collectively these experiences might be best understood as emotional labour, a concept presented by sociologist Arlie Russell Hochschild in her important book “*The Managed Heart*” (Hochschild 1983), referring to an individual’s efforts to regulate emotions in the workplace as may be required to effectively fulfil their roles and duties. Some have suggested that emotional labour is a necessary component of effective human service work, for instance, in the case of healthcare workers in correctional facilities showing compassion for someone who has committed a heinous crime (Guy et al. 2010). Inconsistency between emotion that must be displayed and that which is felt may result in dissonance. This dissonance has been associated with a number of negative outcomes including: self-estrangement or alienation, lowered sense of professionalism, poorer job satisfaction, exhaustion, burnout, and depression (Brotheridge and Lee 2002; Pugliesi 1999).

While affective events related to records with traumatic potentialities and traumatized individuals could indeed be taxing, also notable in the diaries was the joy and satisfaction arising from work with donors, researchers and records. Indeed, participants were more likely to identify heightened happiness and enthusiasm arising from affective interactions with records, donors and users than interactions with others in the organization. As identified in previous research (Aton et al. Unpublished; Regehr et al. 2022) participating archivists described profound satisfaction with the work, arising from the sense that they were contributing to social change, and were helping others find truth and perhaps resolution, at times easing the suffering of individuals. One participant wrote: “I was and still am, so full of joy and love for these dedicated elders”. Others commented: “Upon seeing her thrilled response to finding her family in our database, I was quite happy…I know that this is not just research for them, it’s personal. I know that information they find could potentially be life altering”; “I felt like I was appreciated, helpful, doing the right thing in the right place”.

### Team and leader interactions

While Ashkanasy and Dorris (2017) focus on emotional contagion as a primary source of emotions in group interactions, our analysis of data in the current study focuses more on shared tasks and projects, or where behaviours or actions of group members influence the work of others. For instance, one participant observed:

“I often feel that archives work… is like being in charge of keeping a closet clean, but everyone in your neighborhood will show up unannounced and leave all of their dirty clothes and shoes (and old lightbulbs, etc.) in your closet when you're not looking. It's motivation for improving/standardizing procedures and increasing training, but also just... exhausting”.

Another described their emotional response when a piece of deliberately placed equipment had been moved by a team member: “Anger and frustration. Fairly intense at first (my heart rate going up) and then dissipated as I sat down and tried to think about how I would speak to my director about it.” Another similarly described intense emotion related to a group interaction: “I was gaslit…I honestly was so angry I was shaking.” In another diary entry, this individual described feeling like a “punching bag” and being shown “blatant disrespect”.

On the other hand, group interactions can be highly positive. One participant reported “Joy, excitement, satisfaction, sense of belonging, united” in response to being with others. Another in describing a team problem-solving process reflected: “I felt overwhelmed with gratitude and optimism for what we might accomplish together.” A further participant described moving past a difficult situation as a group, and as a result they “felt really energized and ready to get into deeper and more difficult work that I have been putting off for weeks.”

Ashkanasy and Dorris (2017) suggest that leadership is “a social process that has a major effect on moods and feelings of team members” (p 78). Not only do leaders set the affective tone of a group or workplace, but in addition, a leader’s ability to manage negative emotions events is seen as key component of transformational leadership (Ashkanasy and Tse 2000; Kim and Kim 2017). Research suggests that the behaviour of supervisors and other leaders in the organization can have a significant moderating effect on emotion. For instance, employees in at least one study demonstrate that employees experience fewer positive emotions when interacting with supervisors than with co-workers and members of the public and that supervisors with transformative leadership styles not only provoke more positive emotions in direct interactions between themselves and employees, but also employees and others they encounter in the workday (Bono et al. 2007).

In the current study, 12% of recorded emotional events associated with anger and irritation involved negative interactions with managers, whereas only 2% of emotional events associated with happiness and enthusiasm involved interactions with managers. The negative interactions described were often viewed to reflect a lack of respect for the participant’s knowledge, skills and time, and as noted earlier, provoked feelings that included “anger, frustration and irritation”, anger and defeat, annoyed and irritated, and “quite an emotional outburst…I eventually did start to cry.” (D14) Overall, these interactions were seen to contribute to “Low morale, low value, low sense of valuable contributions to the team”.

### Organizational influences on emotion

Emotional climate of an organization addresses the collective mood of organizational members and how they feel about each other their leaders (Ashkanasy and Härtel 2014). It is a function in large part of an organization’s policies and procedures (Ashkanasy and Dorris 2017). In this study, organizational policies and procedures that influenced emotion fell into several categories. The first relates to overall stances on societal issues as exemplified by two participants’ reflections on their organization’s public stance on the question of abortion and contraception in the aftermath of the court ruling on Roe vs Wade:

“Extremely upset. Hurt, furious, devastated, despairing… the realization that my workplace had a very narrow idea of the “right” response so entirely out of line with my own made this significantly worse”.

“It teaches that contraception, abortion, and medical assistance in dying are not permissible. These ideas run completely counter to the prevailing ethos of Canadian society… The only action is to live with my emotional reaction…and carry on archiving regardless”.

A second set of organizational factors relate to human resource policies and practices:

“We have a new collective agreement, which should be great but everything seems to be going wrong with it. None of us can get clear answers and it's creating so much stress and uncertainty and draining our energy collectively and my energy specifically… I'm feeling so frustrated and exhausted and just tired overall”.

“The toxicity is [organization]-wide… The male bosses are misogynistic and there are occasions when some of the women, particularly the students do not feel safe or comfortable around the male boss”.

Other organizational factors that provoked emotional responses might be best understood as procedures or technologies that impede archival work, such as security access “my colleagues and I were unable to use our badges to access parts of the building”, or inadequate systems “I was consumed by rage at how byzantine and unintuitive it is to find literally anything in any of our archival finding aids”. Another participant reflected “The same type of frustration I feel when I help citizens caught in the bureaucratic meanderings of the [organization] and in the archives when they try to obtain documents they need, and I have to tell them no for bureaucratic reasons.”

The structure and volume of work also contributed to the emotional climate of the organization. People described feeling stressed about workload, and staffing levels, “it’s all rooted in too few staff”, and as a result, at times “the amount of work is overwhelming” and “stress and anxiety are overriding my feelings of joy and excitement for this project”. One person noted “One of my predecessors did not have the proper formation and left a mess—a large backlog—which, 9 years later, I am still victimized by and still cleaning up”.

## Discussion

Building on earlier work on the emotional impact on archivists who engage with records that immortalize human suffering (Nathan et al. 2015; Regehr et al. 2023; Sexton 2019; Sloan et al. 2019), and with traumatized individuals whose lives are reflected in the archives (Douglas and Alisauskas 2021; Regehr et al. 2022; Wexler and Long 2009), the present study sought to better understand the emotional experiences of archivists. Using diary methodology, 15 archivists recorded “emotion generating events” in their workplace (Weiss and Cropanzano 1996) (p 31). Unlike studies focussing on traumatic stress reactions to specific high-intensity events, this approach encourages people to reflect on daily micro-events that influence their immediate emotional state (Junça Silva et al. 2022) and cumulatively influence their work-related attitudes (job satisfaction and commitment) and behaviour (overall performance) (Weiss and Cropanzano 1996).

The diaries of 15 archivists, each of which covered approximately four months of work, reported a wide range of emotions that fluctuated from day to day and that co-existed on any given day, providing data that went beyond that which might be elicited in qualitative interviews seeking to understand a particular set of experiences. More intense emotions of happiness and enthusiasm arose from aspects of the work itself, interactions with records, researchers, donors, and members of the community. On the other hand, more intense emotions of anger or irritation tended to arise in interactions with other members of the organization.

**Fig. 5:**
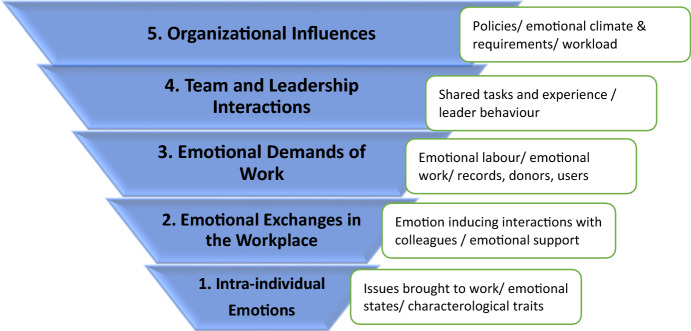
Factors influencing emotional response in archival work. Adapted from (Ashkanasy 2003)

The five-level model for understanding emotions in the workplace developed by Ashkanasy (2003) served as a useful framework for the analysis of the data contained in the diaries. However, during the iterative process of analysis we adapted these categories to better fit the emotional experiences of archivists in this study. The modified model can be found in Fig. 5.

In this new adaptation, *Level 1*—*Intra-individual Emotions*—refers to pre-existing emotional states that arise from other aspects of an individual’s life, these may be related to recent life events (such as a family conflict), to pervading emotional states (such as depression), or characterological traits (generalized optimism). Katherine Miller and colleagues (2007) referred to these as “private emotions”. In this respect, these authors aptly cite Karl Weick (1969) as stating “[w]hole persons aren’t contained in boxes on organizational charts” (Miller et al. 2007), p 237). These individual differences influence how workplace events are experienced and interpreted which subsequently influence emotional and behavioural responses (Beck et al. 1985; Blanchette and Richards 2010; Regehr 2018).

*Level 2—Emotional Exchanges in the Workplace—*focuses on emotional exchanges with others in the organization, primarily co-workers. This level is distinct from the discussions of team and leadership interactions because interactions are based on informal relationships rather than formal roles. Vincent Waldron suggested (Waldron 2000) that workplace *relationships* with colleagues influence emotions more than the *work* undertaken. Daily interactions with colleagues, also referred to as daily micro-events (Weiss and Cropanzano 1996), have the capacity to frustrate or anger individuals cumulatively undermining job satisfaction and commitment (Weiss and Cropanzano 1996). Indeed, colleague interactions were the most common source of anger and irritation among affective events reported in diaries. These daily interactions can serve also as an uplifts, enhancing the overall experience of work (Junça Silva et al. 2022). In this study, uplifts were evidenced by phrases that included gratitude for a co-worker’s contribution, relief through sharing common stressors, and comfort from the support of others. On the other hand, interactions with co-workers can serve as “informal emotional enforcers of organizational culture, sanctioning some emotional displays and discouraging others” (Waldron 1994 p 407). This will be discussed in greater detail as we explore other levels of emotion in the workplace.

*Level 3—Emotional Demands of Work—*has been described by various authors as having varying components, one of which is emotional labour (Hochschild 1979), and another of which is emotional work (Miller et al. 2007). In both these cases, emotions are seen as a *the work* (Hochschild 1979), rather than a *reaction to the work* (Waldron 1994). As indicated earlier, emotional labour is understood as emotion that must be displayed in order to achieve organizational goals. Originally Hochschild studied flight attendants, who must smile and be welcoming regardless of their own emotional state or the behaviour of customers. Archivists in the current study reflected this type of emotional demand when dealing with difficult donors, community researchers, or other community members. In these situations, they described allowing the feelings to subside before answering or brushing the feelings aside. Over time, repeated experiences of dissonance between felt emotions and emotions that are expressed can result in a number of negative outcomes including burnout and depression (Brotheridge and Lee 2002; Pugliesi 1999). Emotional work, in contrast to emotional labour, involves authentic emotion (Miller et al. 2007). The compassion and empathy expressed can at times result in emotional trauma in the worker (Regehr et al. 2022); however, it can also lead to a profound sense of well-being and joy as expressed by diary keepers in this study.

*Level 4—Team and Leader Interactions—*underlines the crucial role that leaders hold in setting the emotional tone of a working group and in supporting and validating workers (Ashkanasy and Dorris 2017; Waldron 2000). Important emotions provoked by leader/employee interactions include pride, fear, and calming relief. Workers become highly attuned to emotional cues displayed by their supervisors and on this basis determine whether to take risks in workplace (Waldron 1994), including innovating and improving processes and services. As has previously been reported (Bono et al. 2007), diary keepers in this study reported fewer positive emotions arising from interactions with leaders, than negative emotions. Often these negative interactions focused on a perceived lack of respect for the skills and knowledge of the archivist. Team member behaviours provoking negative emotions were similarly viewed as disrespectful. Incidents, such as moving a piece of equipment, represented daily micro-events*—*the emotional reactions to which suggested a more pervasive climate within the team. These collective experiences were seen to contribute to low morale and lower team cohesion.

*Level 5—Organizational Influences—*has been described as having two components: (1) the cumulative effects of the other four levels reflecting various affective interactions throughout the organization and (2) overall policies reflecting values and expectations (Ashkanasy and Dorris 2017). In this study, organizational stances on societal issues such as abortion arose from the nature of the organization (for example, religious affiliations), and at times provoked dissonance with personal beliefs of employees. Human resource policies and practices were seen by some to reflect general disrespect of employees. Availability and allocation of resources resulted in perceptions of being overworked. All of these issues formed the foundation within which other affective events were experienced and affected the responses that individuals reported. As noted earlier, organizational factors such as workload, organizational climate, social support and supervision (Bell et al. 2003; Carpenter et al. 2013; Davis-Sacks et al. 1985; McFadden et al. 2015; Sloan et al. 2019) can significantly influence emotional response to affective events. When an organization is perceived to value the contribution of workers and support their well-being, workers are better able to cope with the emotional demands of the job (Alfandari et al. 2022; Elpers and Westhuis 2008).

### Limitations

While the diary method has many strengths, capturing individual experiences temporally close to the time when experiences occur, they also potentially suffer from ecological validity, that is, the degree to which findings can be generalized to other settings or individuals (DeLongis et al. 1992). Further, researchers are only privy to information that the individual has thought to provide at the moment, further contextualization is not possible, leading some researchers to recommend a combination of diary and interview methods (Spowart and Nairn 2014). In this respect, we are not able to situate experiences described within contextual factors of the organization beyond what is deemed important by participants, this is particularly important given the span of organizations represented including religious organizations, communities with shared histories, and governmental organizations rooted in a colonial past. Finally, the use of Ashkanasy’s model for understanding emotions in organizations provided a useful framework for organizing large amounts of data; however, the model was not a perfect fit; thus, we adapted the model, de-emphasizing elements that appeared less salient, such as emotional contagion, and adding new elements such as emotional interactions with donors, researchers and records.

## Conclusion

A recent but growing body of scholarly work is focusing on the emotional toll on archivists of working with traumatizing collections and people who are researching or preserving artefacts of their own traumatic histories (Arroyo-Ramírez 2021; Douglas and Alisauskas 2021; Nathan et al. 2015; Sexton 2019). The increased knowledge in this area is critical (Gilliland and Caswell 2016) and is leading to new educational resources for archivists (Laurent and Wright 2020), and calls for organizational policies and practices that acknowledge and support archivists in this work (Regehr et al. 2023; Sloan et al. 2019).

The current study sought to better understand the traumatic experiences of archivists using a diary method of data collection, seeking to reveal reactions to traumatic events as they occurred, rather than exploring them through retrospective interviews. What emerged from the diaries was a wide range of emotional experiences, arising not only from interactions with traumatizing collections and traumatized people, but more frequently from what have been called daily micro-events (Rueff Lopes et al. 2017), that is, every day interactions that can serve to provoke negative emotional responses or daily uplifts (Junça Silva et al. 2022).

Emotions evoked from encountering affective events are influenced by a number of factors that include: personal issues brought to the workplace, and an individual’s pre-existing emotional state, and characterological traits; emotional exchanges in the workplace including interactions with colleagues; emotional demands of the work (emotion work and emotional labour); team and leader interactions including group tasks and leader behaviour; and organizational policies, climate, resources and demands. We would suggest that this broader set of interactional factors forms the foundation on which traumatic and other troubling events are encountered. Future research must consider the nature of archival organization and interactions within it that contribute to the overall working experience. In addition, given the critical influence played by organizational environments, archival organizations need to take responsibility for creating a culture that demonstrates respect and appreciation for workers, acknowledges the interpersonal challenges of the work, and provides supports for archivists who are shouldering the challenges.
